# The Acquisition Rate and Soundness of a Low-Cost Data Acquisition System (LC-DAQ) for High Frequency Applications

**DOI:** 10.3390/s20020524

**Published:** 2020-01-17

**Authors:** Ciro Moreno, Alejandro González, José Luis Olazagoitia, Jordi Vinolas

**Affiliations:** Industrial Engineering Department, Universidad Antonio de Nebrija, Pirineos 55, 28040 Madrid, Spain; cmorenora@nebrija.es (C.M.); agonzalezmu@nebrija.es (A.G.); jvinolas@nebrija.es (J.V.)

**Keywords:** DAQ, data acquisition system, acquisition rate, low cost, reliability, Arduino, high dynamics, vehicle applications

## Abstract

This article presents a novel and reliable low-cost data acquisition solution for high frequency and real-time applications in vehicular dynamics. Data acquisition systems for highly dynamic systems based on low-cost platforms face different challenges such as a constrained data retrieval rate. Basic data reading functions in these platforms are inefficient and, when used, they limit electronics acquisition rate capabilities. This paper explains a new low-cost, modular and open platform to read different types of sensors at high speed rates. Conventional reading functions are avoided to speed up acquisition rate, but this negatively affects data reliability of the system. To solve this and exploit higher data managing rates, a number of custom secure layers are implemented to secure a reliable acquisition. This paper describes the new low-cost electronics developed for high rate acquisition applications and inspects its performance and robustness against the introduction of an increasing number of sensors connected to the board. In most cases, acquisition rates of the system are duplicated using this new solution.

## 1. Introduction

Data acquisition systems (DAQ) acquire signals from different types of sensors in contact with the physical world. These systems acquire an analog signal and convert it to digital values for further processing. Basically, a DAQ is composed of one or several sensors that send an electrical signal to a circuitry to be conditioned and, subsequently, converted to digital values through an analog-digital converter.

There are innumerable data acquisition systems available in the market. Some of them are based on high priced proprietary systems (hardware and software). The physical magnitude that one wishes to measure significantly conditions the acquisition system to be used. It is important to determine if the measure you want to acquire changes over time and at what frequency. There are physical systems with magnitudes that change slowly with time (for example the temperature) and other systems in which they vary many times per second. The physical system to be measured, therefore, determines the characteristics of the acquisition system that can be used to provide reliable data.

In this article, we focus on a very demanding application, the measurement of dynamic magnitudes in vehicular systems. This application needs to quickly process high frequency signals coming from the sensors installed in the vehicles (displacement sensors, acceleration sensors, GPS sensors, inclination sensors, etc.) in front of external solicitations (inertias, vibrations, change of position, speeds, accelerations, etc.). The configuration of an acquisition system compatible with these requirements causes the price of the data acquisition system to skyrocket. Additionally, the final price depends on the number and quality of the sensors used, as well the refresh and data acquisition rates must be high which means that the processors integrated in the circuitry must have a powerful and quick processor. At the same time, the system should store and record the acquired data without delay.

Given this situation, we recently introduced an alternative low-cost acquisition system based on the Arduino platform [1]. In that study, we validated the low-cost system presented with a professional data acquisition system for vehicular dynamics applications. The results were promising, validating the use of an Arduino-based system for this type of applications with high reliability at a reasonably high sampling rate when a reduced number of sensors were installed. 

The idea of using a data acquisition system based on a low-cost platform is not new. In [2], Arduino had already been presented as a possible low-cost laboratory equipment and its accuracy was analyzed, although an investigation of its performance and capacity was not studied or taken to the limit. Other acquisition systems based on low-cost platforms, such as Raspberry Pi, have also been presented, as in [3], where the system was used as a data acquisition and storage system where large amounts of sensor data on low-cost servers was stored.

The relevant literature shows that these low-cost platforms have been applied to countless situations. Most of these applications do not need high frequency of acquisition or do not apply to very demanding dynamic situations. For example, in [4], a low-cost system was developed based on an Arduino Mega platform that deployed a variable gain amplifier circuit increasing the efficiency, resolution, and measuring range of the system. In this case, the system was also connected to a Raspberry Pi with the calculation of basic electrical parameters in real time. Another recent example was the development of an automated calorimeter for determining soil-specific heat using a microcontroller based on Arduino [5]. It was also used for acquisition of environmental data, as in [6], where a system based on an Arduino Leonardo system was used for continuous monitoring of air quality. Another significant example was the use of a KdUINO DIT system to estimate water quality through the study of its transparency through the diffuse attenuation coefficient [7] that was recorded on an SD card for further study. 

Some applications can be found in which these low-cost systems have been applied to high-frequency dynamic systems. For example, in [8], a low cost acquisition system was developed for a single axis vibration shaker table for civil engineering applications. In this case, the shaker table was used to simulate horizontal acceleration data recorded during earthquakes and was based on an Arduino DUE for control of the table and an Arduino MEGA for the verification of the acceleration obtained. Another study obtained dynamic data in a low-cost platform to study the lateral displacement of railway bridges through the use of low-cost accelerometers transmitting the data wirelessly [9]. Another recent example is found in [10], where a real-time electronic system was presented to monitor the integrity of structures, continuously monitoring data such as acceleration, inclination, position, and temperature that are transmitted wirelessly using the harvested energy in the system. It is also possible to find applications in which these low-cost systems are used to condition the operation of a machine. For example, in [11], a low-cost system was developed to monitor a bearing in a mechanical system through a MEMS accelerometer, transmitting the information to a computer for further processing in Matlab. Other applications have used multipurpose equipment (such as National Instuments DAQ) [12] to lower the cost of high dynamics (and higher priced proprietary systems), such as photoacoustic computed tomography [13].

In regards to the low-cost acquisition for applications related to vehicles, in [14], a low-cost system was used to assess the comfort of travelers in the public transport system while evaluating vehicle accelerations in a determined route by urban route. This system used a compact system obtaining data from a GPS sensor and an accelerometer. In [15], a DAQ and a driving operation process was designed for a dynamic vehicle simulation system, where data of the path of the three driving pedals were obtained through linear displacement sensors and a sensor of angular displacement to obtain the steering angle of the steering wheel. In [16], it was possible to classify the type of behavior behind the wheel of public transport drivers using of a single low-cost triaxial accelerometer. The same idea was applied in [17] using an inertial sensor and the CAN bus. In addition, in [18], an analysis of cyclists data obtained by sensors was carried out through an Arduino-based system, using an ultrasonic sensor and a GPS to evaluate the type of roadway. The system connected to a smartphone via Bluetooth to record the data on a server. Recently, a low-cost system was presented, in this case based on Raspberry Pi, to assess road surface friction [19]. In this case, the deceleration from the braking of the vehicle was measured and compared with the geolocation of the vehicle to calculate the corresponding coefficient of friction.

The examples cited so far prove that it is possible to use low-cost acquisition systems in increasingly demanding situations. However, the specific applications in which Arduino- or Raspberry Pi-based systems have been used for vehicular dynamics applications are still limited. In the system presented in a previous article [1], we already have shown that it was possible to dynamically use a system based on an Arduino microcontroller to perform dynamic measurements in vehicles. However, little attention has been given in the relevant literature to improving the sampling frequency and the reliability of these low-cost systems. The articles consulted are limited to highlighting the feasibility of acquiring, analyzing, or sending data through low-cost platforms in specific applications, but little is explained about reliability or how to increase the acquisition rate on these systems.

In reality, to implement a low-cost acquisition system in these platforms, it must be taken into account that the system’s control, the testing, and the error checking are not implemented by default. In addition, no information is given about the reliability of the obtained data or on how to improve the acquisition rate of the system. Addressing the available functions in a low-cost acquisition system in order to further speed it up forces the user to be prepared to deal with different types of errors. The transmission of the data could have errors that should be monitored for a reliable acquisition. If the DAQ do not have an integrated and adequate error checking system, the costly experimental results would have to be repeated and, consequently, with a waste of time and money. In applications such as dynamic acquisitions in vehicles, this is especially relevant, since the availability of these systems to carry out tests is usually limited. In professional acquisition systems, many of these errors are controlled and the system automatically corrects for them. In low-cost platforms, this control system is usually not available, and one has to program for it.

A low-cost acquisition system for high dynamic applications must not only guarantee obtaining proven reliable data, but it must also assure high acquisition rates. In addition, it is desirable that the system is easy to use and configure, modular, and reusable in different vehicle configurations. Unfortunately, little attention has been given to improving acquisition rates and the errors that can be generated in these Arduino-based acquisition systems and how the data should be treated to ensure fast, continuous, nonstop, and reliable operation.

Taking the system used in dynamic applications described in [1] as a reference, it would be desirable to increase the already high acquisition rate provided. In addition, communication through the serial ports between the system of Arduino (that provides the measure of the real time) and the central computer (that can be based on a Raspberry Pi in case of wanting to maintain the cost low) can have transmission errors that one has to monitor. 

As a result of the analysis of the revised literature, we concluded that the development of applications based on low-cost electronic platforms is increasing and is very popular. However, the use of these platforms for the acquisition of high-frequency dynamic data is limited. Applications that use these platforms for data acquisition in dynamic vehicle applications are much more restricted. In addition, to the best of our knowledge, none has directly addressed the improvement and optimization of the speed of data transmission on these platforms to improve their operation.

Therefore, the scientific novelty of this paper is centered on the development of a novel DAQ, based on affordable electronics for real-time applications in vehicular dynamics that, apart from allowing the open installation of a wide variety of sensors, multiplies by two the acquisition rate of the system presented in [1] and assures consistent reliability of the acquired data, checking and automatically correcting the detected errors. Specifically, this new system includes the configuration of new hardware, with a redesigned shield adapted to be modular and open to innumerable types of analog and digital sensors (position sensors, accelerometers, GPS, etc.). In addition, the transmission of reliable (and time-marked) data from the sensors to the central operating system is assured. Optionally, the system can work autonomously recording the acquired data in a local SD card. The reliability of the data acquisition system is completed with a real-time data analysis module that detects and corrects situations in which errors occur, making the system functional and fault tolerant. Therefore, a robust acquisition system is proposed, which allows reliable professional acquisitions at a low cost. In addition, the system is easy to install and very small, aimed at use for dynamic applications in different vehicle sizes.

This article is divided into different sections. Section 2 details the characteristics of the new acquisition system. In this section, first, the hardware configuration is described and, secondly, it deals with the study and improvement of the reliability of the operation of the low-cost hardware used and the protocols that must be implemented to ensure the quality of the data and its transmission at a high refresh rate. Section 3 focuses on the validation tests of the system and presents the system functioning in a real application, as well as a discussion of the results obtained. Section 4 presents our conclusions and future paths. 

## 2. The Low-Cost Data Acquisition (LC-DAQ) System

The low-cost data acquisition system presented in this research is aimed to conform an open, flexible, robust and low-cost system which acquires data at high frequency from a large number of sensors simultaneously, providing high accuracy on the data acquired. 

On the one hand, Arduino is a good platform for the development of such a system. It is fully open source, and the development boards that are compatible with it are electrically reliable and available at a low cost. Furthermore, there is wide flexibility in terms of connections and digital protocols that can be achieved with this platform. Thus, this becomes a suitable choice to develop the hardware of the LC-DAQ system.

On the other hand, the serial communication protocol between the Arduino boards and external computers becomes a bottleneck in terms of sampling frequency in real-time acquisitions applications. The Arduino’s built-in functions for communicating with the controlling PCs are robust but do not focus on efficiency. In order to increase the acquisition frequency and to optimize the robustness of the system, a custom communication protocol is developed and introduced as part of the LC-DAQ system.

### 2.1. Hardware

The proposed low-cost system, namely low cost data acquisition system (LC-DAQ) is a modular system with a high capacity for personalization. It is optimal for vehicle data acquisition applications where compact and reliable systems are necessary. The LC-DAQ is composed of an Arduino Due card. The board performs the processing of the data. An acquisition Shield (developed in the present work) simplifies the connections between the sensors, modules, and the acquisition card. Data can be stored directly on the SD card allocated in the shield, or it can be sent online to an external computer. The system is protected by a housing made of methacrylate. 

Arduino Due has a powerful Atmel SAM3 × 8E ARM Cortex-M3 with a 32-bit CPU. This provides a faster calculation and acquisition speed than boards based on AVR architecture. Arduino Due is a device far superior to previous models. It has 54 digital inputs and outputs, of which 12 can be used as pulse-width modulation (PWM) outputs. It has 12 analog inputs and two analog outputs with a voltage range between zero and 3.3 V. Four serial ports through the hardware (UARTs) establish several serial connections at the same time. It also has pins dedicated to the I2C (inter-integrated circuit) and SPI (serial peripheral interface) protocols. Table 1 shows the characteristics of the Arduino Due card as compared with other Arduino compatible systems. As it can be observed, the Arduino Due sports the highest clock frequency and finest analog resolution. Along with its low supply voltage (3.3 V), these two features represent fundamental advantages over other systems to fulfill the requirements of the LC-DAQ.

**Figure 1:**
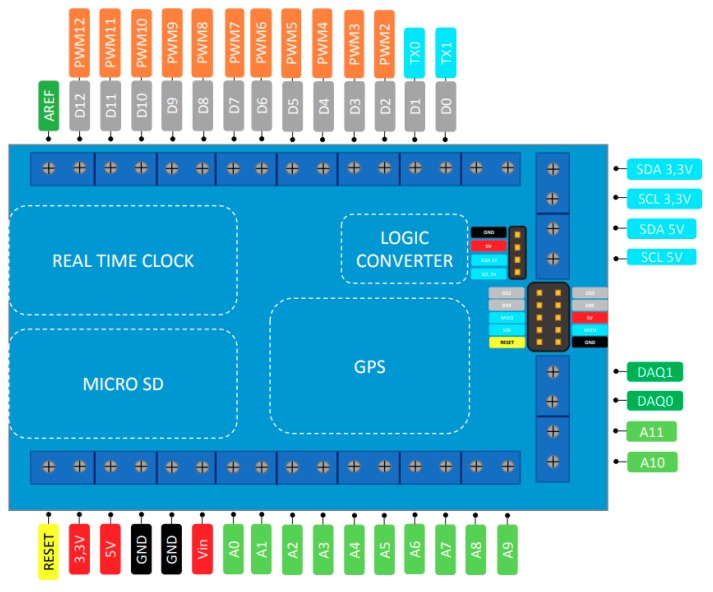
Low-cost data acquisition (LC-DAQ) shield pinout.

A customized shield (Figure 1) is easily installed on the Arduino Due board. The shield connects the different modules and the external sensors. Optional SD card, GPS and real-time clock (RTC) modules can be installed on the system. The shield has female pins to directly connect modules to the acquisition system. It is a Plug and Play system. External sensors such as accelerometers and displacement sensors are connected by terminals. The acquisition shield connects the output and input pins of the Arduino Due board to external terminals. The terminals have a screw fixing system. The external sensor cables are connected to these terminals and thanks to this, system reliable connections between the elements are guaranteed. This construction allows the data acquisition to be easily embarked in the vehicle. Connection problems due to vibration are avoided. In addition, it is a customizable system with fast assembly. The available pins are shown in Figure 1. The optional modules are installed depending on the application and the available budget. 

**Figure 2:**
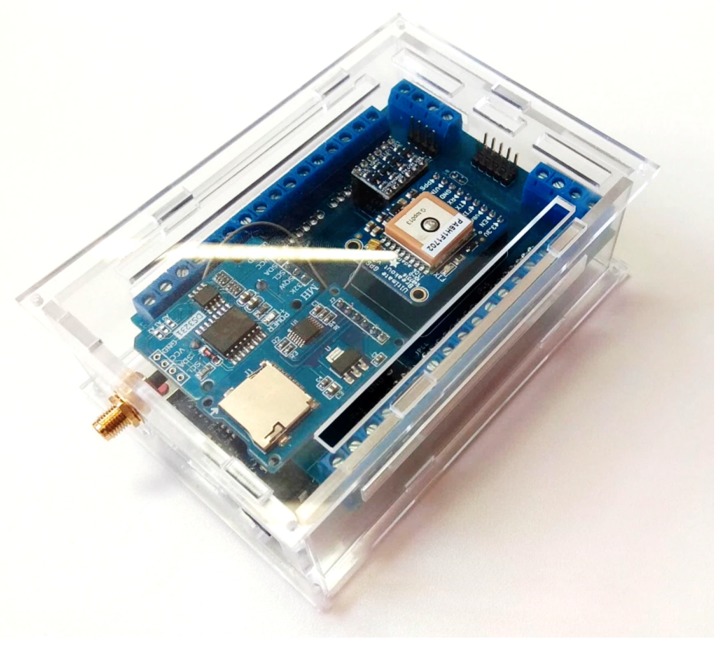
LC-DAQ system with all the modules installed.

The shield has four different optional modules. The first module, the microSD module Catalex v1.0, is used when acquisitions are made using the acquisition system individually, that is, without an external computer. The module is connected through the SPI protocol and the SD acquisition allows a compact system but limits acquisition frequency to 70 Hz [1]. The second module, the RTC module DS3231, is connected through I2C and the watch allows us to have control of the time for long-term acquisitions. In short acquisitions the clock of the microcontroller is used. The third module has a GPS module Adafuit Ultimate V3 which allows the position and speed of the vehicle to be known with frequencies in the range of 1 to 10 Hz. Its speed accuracy is ± 0.1 m/s and the position accuracy is ± 1.8 m. Finally, the fourth module is a logical converter for the I2C protocol. This is needed to use sensors that operate at 5 V in the LC-DAQ. The LC-DAQ system is shown in Figure 2.

The acquisition system supports three forms of connections for external sensors (Table 2). Analog, I2C (inter-integrated circuit) and SPI sensors can be used. The voltage range of the analog pins is from 0 to 3.3 V. Negative voltages cannot be measured. Any sensor able to either communicate through one of these protocols or to return an analog signal within the voltage limit is compatible with the system.

The analog digital converter of Arduino Due has 12 bits. Therefore, the resolution is 0.806 mV for a voltage range of 0 to 3.3 V. This obliges to condition the signal from sensors that do not meet these characteristics using voltage dividers.

The sensors that are used (MPU-6050 triaxial accelerometers) communicate through the I2C protocol which is preferred because it connects up to 255 sensors using only two cables. These two cables are the serial clock line (SCK) and the serial data line (SDA). Both lines are connected to all the devices that are using the data bus. The devices that use this protocol can be masters or slaves. In this case, the LC-DAQ is the master and the external sensors are the slaves. The LC-DAQ system controls the clock line and data transfer is only started by the LC-DAQ. The system supports connecting I2C sensors powered at 5 V or 3.3 V. An example of the sensors that use this connection are MPU6050 accelerometers, the LCD screen, and the RTC.

Finally, there are sensors that communicate through the SPI protocol. This communication protocol is characterized by being faster than I2C. In addition, it does not need a dedicated address to identify each sensor. It uses four wires, a serial clock (SCLK), a master output slave input (MOSI), a master input slave output (MISO), and a selection pin (SS). The microSD Catalex V1.0 module uses SPI for the data storage.

The power consumption of the LC-DAQ is 80 to 90 mA plus 4 mA for each connected sensor. Therefore, without additional modules, the consumption of the LC-DAQ together with eight triaxial accelerometers MPU-6050 is approximately 112 to 122 mA.

### 2.2. Firmware and Communication Protocol

The firmware installed into the LC-DAQ is developed taking advantage of Arduino IDE. The LC-DAQ is meant to be a robust and affordable acquisition system that is able to deliver a high enough sampling frequency in a real-time scheme. This means that LC-DAQ must be connected to a master computer through a reliable and fast communication protocol. Serial over USB is the most commonly used communication protocol between Arduino boards and computers. Arduino IDE provides defined functions to send and read data packages through the serial port. One of these functions is Serial.print. It becomes very convenient to send data in a structured and reliable manner. In a previous version of LC-DAQ, Serial.print was the default function used to communicate with the external computer [1]. Serial.print function is a regular and reliable communication function. However, it does not allow high sampling frequencies, especially when several sensors are connected to one board. 

The speed in the serial communication can be improved in two ways. On the one hand, the serial port can be configured to operate at different baud rates. On the other hand, the communication protocol defines the number of bytes that are needed to send the acquisition information. The volume of these bytes directly impacts the communication speed and the sampling rate. By improving the communication protocol, the communication speed can be significantly improved. This research focuses on improving the serial communication protocol to enhance the acquisition frequency. In order to test the improvements of the new protocol, the baud rate was set to a commonly used value of 250,000 bps. 

A qualitative explanation can be provided observing how Serial.print works. This function takes the variable specified by the user and converts each of its characters into ASCII code and, then, sends a byte containing this code for each of the character in the variable. This is reasonable for sending text. However, sending numbers under this scheme becomes totally inefficient for LC-DAQ, which is meant to read numerical values from different sensors and timers and transmit them to a master computer in real time. As an example, consider that one of the values is the number 255 in base 10. In Hexadecimal this data is represented as 0 x FF and in binary it becomes the single byte 11111111. Using Serial.print (255) this value, first, is split into its three characters [2, 5, 5]. Then they are converted into ASCII code, which in Hexadecimal is written as [0x32, 0x35, 0x35] and in binary as [00110010, 00110111, 00110111]. Then, these three characters are sent individually as three bytes embedded into the communication protocol used by the function to guarantee the data synchronism and reliability. Under this scheme, the number of bytes that must be sent is multiplied by three. Furthermore, the user has no control of the communication protocol that runs every time that a Serial.print command is invoked.

In order to overcome the data size disadvantages of using Serial.print, this work embodies the integration of a communication protocol over serial port that drastically reduces the volume of data that has to be sent and notably increases the acquisition frequency obtained with the LC-DAQ system. However, avoiding the use of Serial.print, which is reliable, means that the control and reliability of the communication rely on the user. This protocol pivots on one of the functions provided by Arduino IDE (Serial.write) which sends bytes individually and grants full control to the user. This protocol is assembled adding different control layers to improve the speed and robustness of communication. These layers have been tested individually to isolate their effects on the transmission speed and data accuracy. Statistical analyses were performed at each step of the study tracking all the different errors appearing on the communication to ensure the reliability of data and to observe the pros and cons of each control layer added to the protocol as follows:

- **Data framing** Increases transmission speed by sending bytes instead of ASCII characters;
- **Checksum** Increases robustness by detecting errors in the byte chain;
- **Time resolution reduction** Increases transmission speed by reducing the number of bytes in time variables;
- **Bytes stuffing** Increases robustness by including control bytes to prevent interpretation errors in the receiving algorithm.

#### 2.2.1. Data Framing

This first layer uses Serial.write instead of Serial.print. It also implements data framing to synchronize the data flow in the transmission. Serial.write receives the following two arguments: The address of the variable to be sent (pointer to the variable’s first memory address) and the number of bytes to be sent (number of memory positions to be read and send through serial port starting at the memory address introduced in the first argument). The variable that has to be transmitted is stored in the memory as a sequence of bytes. If the number of bytes indicated in the second argument of Serial.write exceeds the length of that variable, Serial.write would continue sending those bytes stored into the contiguous memory addresses. Consequently, data must be stored carefully structured into the microcontroller memory, and therefore the program has full control on the data location. 

In order to control the data, different structured variables are defined for two kind of sensors, i.e., analogic and digital. The variable for analogic sensors stores the following two variables: the time count read from the timer at each measure and the digitalized value of the voltage returned by sensors’ transducer. The variable for digital sensors can store several values, i.e., the time value (uint32_t) and as many digital values (uint16_t) as the sensor is able to send at each measurement. For example, the MPU6050 used in this study is configured to return up to three accelerations from the sensor.

On the one hand, with a resolution of microseconds, 32 bits allow the sensor to register continuously up to 1.19 h. This time is enough to carry out most of the experimental tests for which this LC-DAQ system was conceived. On the other hand, the analog to digital converter (ADC) of Arduino DUE has a native resolution of 10 bits. This can be increased up to 12 bits of real resolution and extended up to 16 bits by interpolation. In addition, all the digital sensors used with our LC-DAQ system have a resolution of 16 or less bits. 

This approach stores all the data of one sensor in a single structure which is allocated sequentially, byte-by-byte, in the memory of the system. Being so, the stored information of one sensor can be send just by calling the function Serial.write with the pointer to the structure defined for that sensor and the total length of the data stored on it (six bytes in the case of an analogic sensor) as arguments.

**Figure 3:**
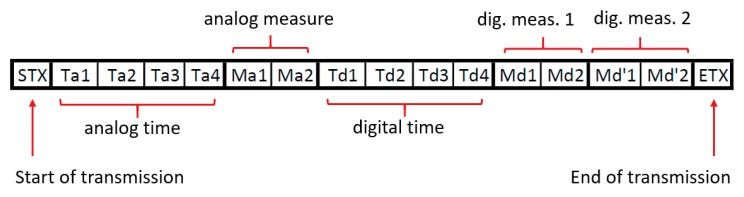
Example of bytes string enclosed between the special characters of start of transmission (STX) and end of transmission (ETX). Each block represents a byte. Each variable contains several bytes which are sequentially sent staring at the less representative one.

Framing characters are used to create a data frame within the string of bytes transmitted which synchronizes the data flow between the LC-DAQ and the master PC. These characters are represented by the start of transmission byte (STX) and the end of transmission byte (ETX), typically 0x02 and 0x03, respectively. Figure 3 shows the sequence of bytes sent through the serial port to transmit the information of one analogic sensor and one digital sensor with two variables.

Data is sent from the LC-DAQ enclosed within the framing bytes. That information is received in the PC, where a Python program monitors the serial port and classifies the incoming bytes. Once it reads a STX byte it stores the following bytes in its memory until an ETX byte is received. Then, it counts the number of bytes received and if it is equal to the number of bytes expected, it reconstructs the values of the variables from the bytes received and performs the conversion of those values into the equivalent physical quantities. If the number of bytes stores does not coincide with the expected one, those bytes are discarded, and a new chain of bytes is read.

Therefore, framing drastically reduces the number of bytes in the serial transmission for each set of measures performed by the system, increasing the sampling frequency (see the Results section). 

#### 2.2.2. Checksum

The checksum layer is intended to improve the consistency of the data transmitted. It performs a checksum operation on the byte string before it is sent (in the LC-DAQ side) and after it is received (in the PC side). Then, both values are compared, and data is approved if they coincide. 

**Figure 4:**
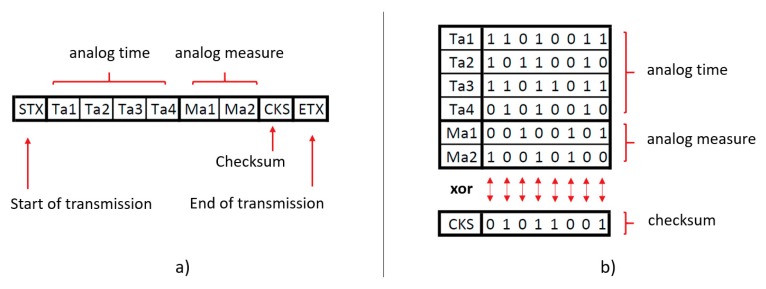
(**a**) Example of a string of bytes containing the data of an analog sensor along with a checksum byte and (**b**) numerical example of XOR checksum calculation.

The checksum performed at both sides (LC-DAQ and computer) consists in a byte-by-byte XOR logical operation. In this way, the probability of errors cancellation is reduced, obtaining eight parity bits for the full string. Figure 4 is presented as an example in which the information of an analog sensor is transmitted. Figure 4a shows the string of bytes, whereas in Figure 4b a numerical example of the bytes contained in the string is presented along with the result of performing a XOR checksum on the six bytes corresponding to the data.

#### 2.2.3. Time Resolution Reduction

This layer is meant to further reduce the number of bytes to be sent in the string of bytes for each communication. Initially, the resolution of the time value for both analog and digital sensors is 32 bits. These four bytes allow the sensor to codify up to 71.5 min in intervals of 1 microsecond. By reducing the time resolution to 24 bits (3 bytes) the maximum time period that can be codified reduces to 16.7 s. This time span becomes too short for most of the experiments for which LC-DAQ is designed, but it is long enough to allow the execution of several hundreds and thousands of program’s main loop in the Arduino side, depending on the number of sensors to be read.

To ensure the integrity of the time count, a time-correction routine is included in the PC side that stablishes the initial time and sequentially adds the time increments detected at each step, by reading the raw data provided by the Arduino timer. In the first reading, this routine stores the initial value of time received on the serial port, and therefore it can subtract this value from the consecutive values of time and set the time of the initial measure as zero. At each reading, the time-correction routine compares the value of time received in the current reading with that received in the previous reading. Then, it adds this difference to the previous value of time computed. At a certain number of readings, the maximum value of time that can be stored in the 24 bits variable is reached and the time’s count is reset. Then, the value of time received on the serial port is lower than that in the previous reading. When this circumstance is detected, the comparison between the current value of time received with the previous one becomes a negative value. The actual time interval that elapsed between these two measures is found by adding the maximum value reachable in the time variable (224 = 16.777.216 µs) to the times difference. By this approach, the maximum time achievable at any experiment becomes virtually infinite.

**Figure 5:**
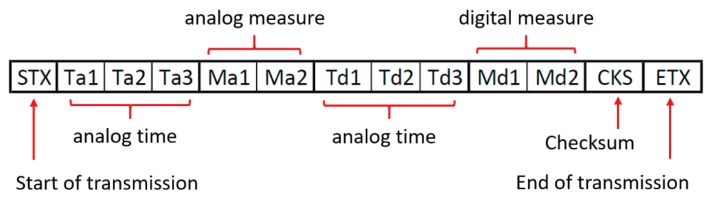
Example of a string of bytes containing the data of an analog sensor, a digital sensor of one variable, and the checksum byte. The time resolution has been set to 3 bytes (24 bits) for both sensors.

It would be possible to further reduce the number of bytes used to encode the timer count. For a timer count with 2 bytes of resolution with sampling times of 1 microsecond, the maximum time that can be encoded is about 65 ms. However, as far as the number of sensors is incremented, the time taken for the Arduino program to perform a single main loop becomes closer to this value. At a certain point this circumstance could produce aliasing on the different measures, therefore, we set the time resolution in 3 bytes as a compromise between performance and robustness. Figure 5 shows how the data string would be sent at this stage.

#### 2.2.4. Stuffing

The flags (STX and ETX) are special characters that identify the start or end of transmission and are codified with a single byte. Table 3 presents the codification used for some special characters. 

**Figure 6:**
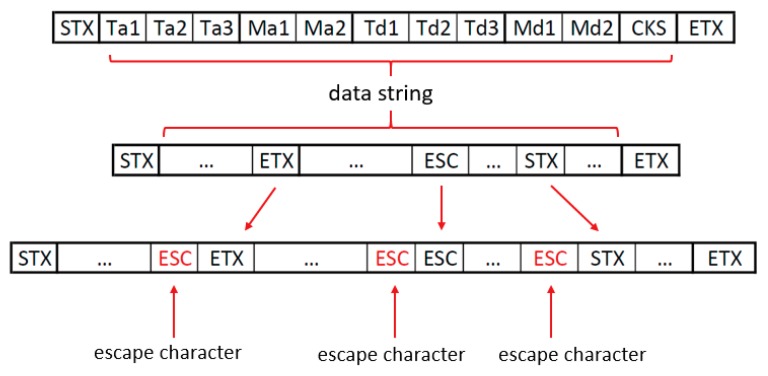
Example of byte stuffing on a data string containing three data bytes that coincide with the special characters.

The data framing layer (see Section 2.2.1) is effective when the bytes within the data string are different from the special characters. However, if some of te bytes that codify either the times or the measurements takes the same value as one of those special characters, the system which receives the data string (the master PC in our case) could misinterpret the framing structure of the message and result in a reading error. To prevent the system failure, a byte stuffing technique is used. This is an extended technique in serial communications which consists of introducing an extra character (ESC) before those data bytes which coincide with other special characters. When the receiving system reads an ESC byte it knows that this character must not be saved and that the following byte must be treated as data instead of as a special character. Figure 6 shows an example in which some of the bytes contained in the data string coincide with the special character and how the communication protocol modifies the string by stuffing the ESC byte.

The four techniques presented above develop a communication protocol which provides full control of the data transmitted, highly increases the communication speed by reducing significantly the volume of bytes sent, and provides a high level of robustness on the data transmission. 

In the following sections, the effects of introducing such control layers into the communication protocol are experimentally studied observing the sampling frequencies achieved by LC-DAQ (configured with different number of sensors), as well as the time regularity obtained in the measures. 

## 3. Methods, Tests, and Results

For the evaluation of the different data transmission methods, 5 trials were carried out. The LC-DAQ system was used together with low-cost triaxial accelerometers. Measurements were taken with different numbers of sensors and 1 to 8 sensors were connected. A time measurement was taken for each acquired sensor and 1, 2, or 3 axes were measured at the same time for each sensor. Only one “time” value was acquired per sensor. Therefore, the number of time bytes sent in each cycle depended on the number of sensors and not on the number of axes sampled. The maximum acquisition frequency was analyzed for the different test methods. Errors produced in each test were recorded. The study was divided into 5 tests as shown below.

Test 1 was the baseline. It consisted in the acquisition of acceleration values provided by a different number of MPU6050 triaxial sensors. The test was divided according to the number of axes acquired at the same time (1, 2, or 3) and 1 to 8 sensors were acquired simultaneously. The transmission of the data was implemented with the Arduino’s Serial.print function. On the receiving PC, a Python program was executed to monitor and record the time at which the data was received. In this test, only the maximum acquisition frequency is obtained. It is not possible to register the number of communication errors. The relative standard deviation (RSD) of the frequency is used as a representation of the number of errors. When there was an error in a measurement cycle, the sensor data was not recorded. With a bigger number of errors there are more frequency drops. The test was performed in static way. In this situation, the value of bits sent by the sensors was constant.

In Test 2, the same tests were carried out implementing data framing. This reduced the number of bytes sent and kept track of the errors produced in the transmission. The test was performed following the same methodology as in the first test. With the data framing method, it is possible to know if there are errors in the structure of the data sent. Therefore, a record of the errors was taken in a .log file and 1 to 8 sensors with 1 to 3 simultaneous axes were sampled. The maximum frequency values and errors were recorded. This method sends the same number of bytes regardless of the value sent by the sensors. Therefore, tests could be made static or dynamic without varying the deviation from the frequency of acquisition. This behavior is different with the Serial.print method.

In Test 3, checksum was integrated to increase the robustness of communication between the LC-DAQ and the PC. The same procedure was followed as Test 2. Data acquisition frequency and structure string errors were recorded. The introduction of checksum allowed the byte corruption errors to be known. The addition of one byte per string sent was studied.

In Test 4, time resolution reduction was included, reducing the number of bytes of the time variable (3 bytes instead of 4). This change was implied by sending one less byte for each acquired sensor. The methodology followed was the same as the two previous trials. Acquisition rates were sampled, as well as the error log.

Finally, in Test 5 the stuffing layer was implemented. The experiment followed the same procedure as the previous tests. Acquisition frequency and string structure errors were logged. The results obtained in the experimental trials using the LC-DAQ low cost acquisition system are shown below.

**Figure 7:**
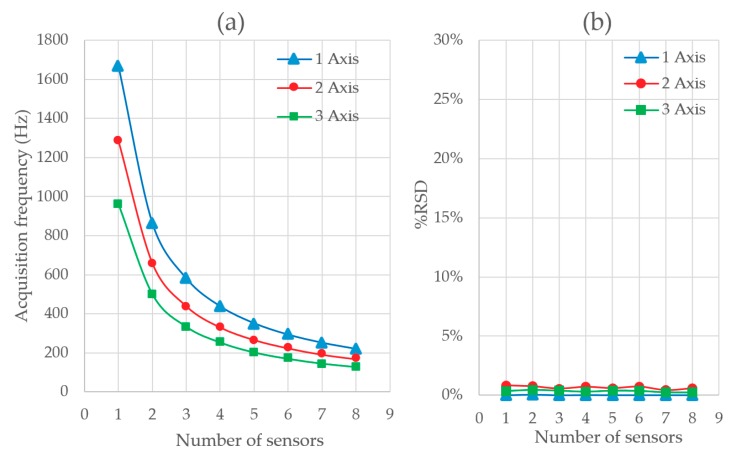
(**a**) Acquisition frequency depending on the number of sensors and axis read at the same time with Serial.print method and (**b**) acquisition frequency relative standard deviation (RSD) depending on the number of sensors and axis read with Serial.print method.

Figure 7 shows the results obtained in Test 1. In Figure 7a the acquisition frequency is represented versus the number of sensors sampled by the Serial.print data acquisition method. In the ordinate axis, the acquisition frequency is shown in each test. The number of sensors acquired at the same time is represented on the abscissa axis. The three curves represent the I2C triaxial accelerometer sampling 1, 2, or 3 axes at the same time. The nonlinearity of the acquisition frequency can be observed. The maximum frequency reached is 1666 Hz for a sensor acquiring on a single axis. The minimum acquisition frequency is 127 Hz for 8 accelerometers acquiring on 3 axes. The greater the number of axes sampled, the lower the acquisition frequency. The speed decreases between 23% and 18% when an additional axis is included. Figure 7b shows the relative standard deviation (RSD). The RSD is a standardized measure of dispersion of a frequency distribution. In this case, it is directly influenced by the number of errors in communication. Each error represents a discarded measure and implies that a new program cycle is necessary to obtain a correct measurement. This doubles the acquisition time when there is an error. With this method we obtain a deviation of up to 0.8%. Therefore, it is observed that Serial.print has some errors filtered but it is not possible to identify them.

**Figure 8:**
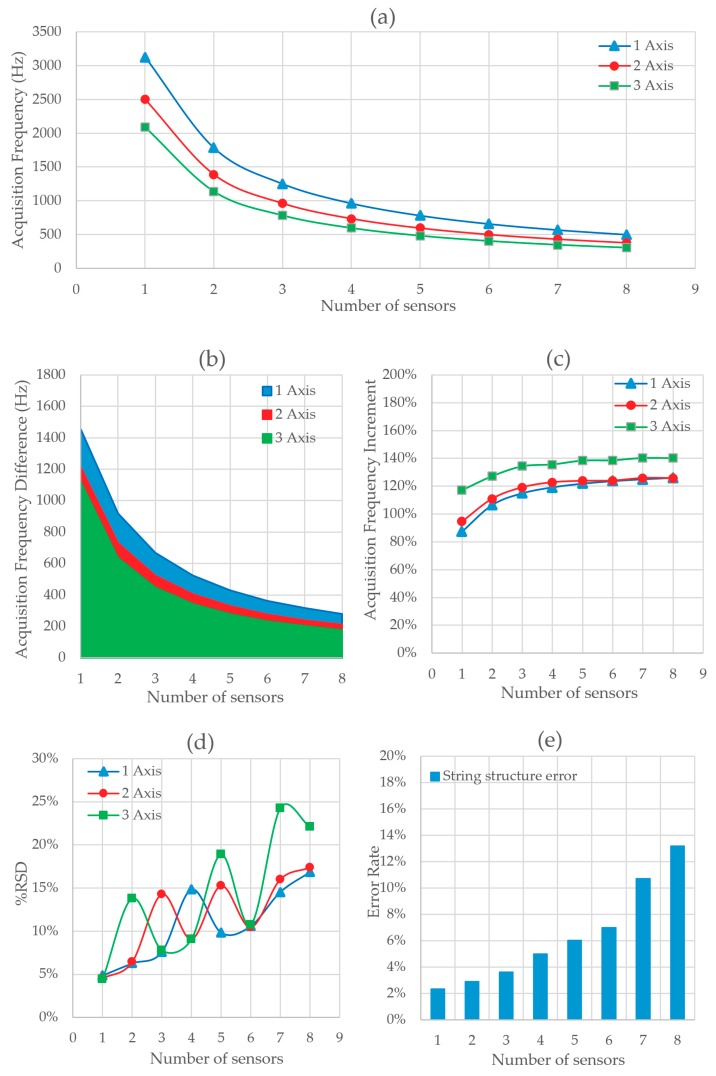
(**a**) Acquisition frequency depending on the number of sensors and axis read at the same time with data framing method, (**b**) total variation of the data acquisition frequency between Test 1 and Test 2, (**c**) percentage increase of the data acquisition frequency between Test 1 and Test 2, (**d**) acquisition frequency RSD depending on the number of sensors and axis read with data framing method, and (**e**) total error rate in Test 2.

Figure 8 shows the difference in results obtained between Test 1 and Test 2. In this Test, data communication between the LC-DAQ and the computer is done by sending byte string and not by the Serial.print method. Figure 8a shows the evolution of the acquisition frequency as a function of the number of sensors and axes acquired at the same time. The frequency is increased by 1458 Hz in the best case. Figure 8c shows the increase in the relative acquisition frequency between Test 2 and Test 1. Very significant improvements in the acquisition frequency from 80% to 140% are obtained. The increase in the sampling rate is due to a reduction in the sent bytes. It is observed that the increase in speed is greater when the number of sensors or axes acquired simultaneously is increased. This happens because the number of bytes sent is greater in each data string, the improvement of the new method is proportionale to the numer of bytes sent. The relative deviation in the acquisition frequency is shown in Figure 8d. We can see that the measures discarded by this method are greater than in Test 1. Figure 8e shows the errors found in the .log file. 

**Figure 9:**
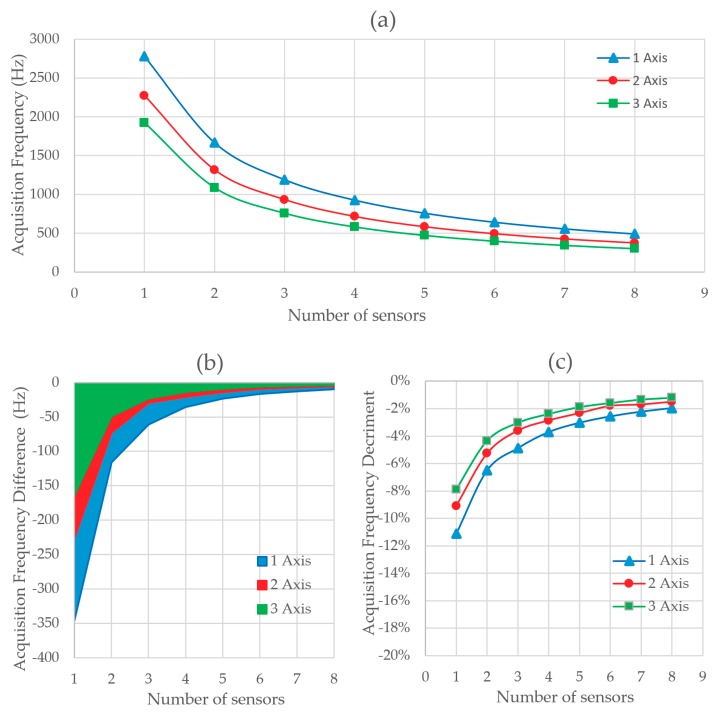

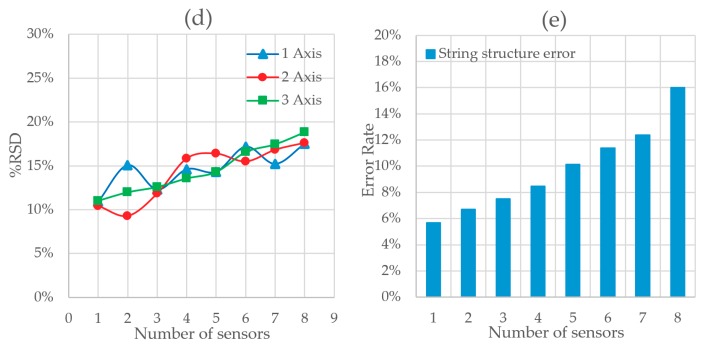
(**a**) Acquisition frequency depending on the number of sensors and axis read at the same time with Checksum method and 4 bytes of time, (**b**) total variation of the data acquisition frequency between Test 2 and Test 3, (**c**) percentage increase of the data acquisition frequency between Test 2 and Test 3, (**d**) acquisition frequency RSD depending on the number of sensors and axis read with Checksum, and (**e**) total error rate in Test 3.

The result of Test 3 is shown in Figure 9. Figure 9a shows the acquisition frequency versus the number of sensors acquired. A decrease in the acquisition frequency is observed as compared with Test 2. This difference is observed in Figure 9b and in Figure 9c, where the absolute and relative difference of the acquisition frequency are, respectively, represented. The acquisition frequency decreases to a lower extent when the number of sensors or measurements is smaller. This is because the sending of the checksum byte has a greater influence on time when the number of bytes of data sent is smaller. The acquisition frequency for 1 MPU6050 measuring 1 axis is 2770 Hz. With 8 sensors taking a measurement the frequency decreases to 490 Hz. The relative standard deviation of the frequency (RSD) is between 10% and 20% and is represented in Figure 9d. Finally, the errors recorded are shown in Figure 9e. Under laboratory conditions no checksum errors were found during the tests. The most frequent error is still sending byte strings that are too short. 

**Figure 10:**
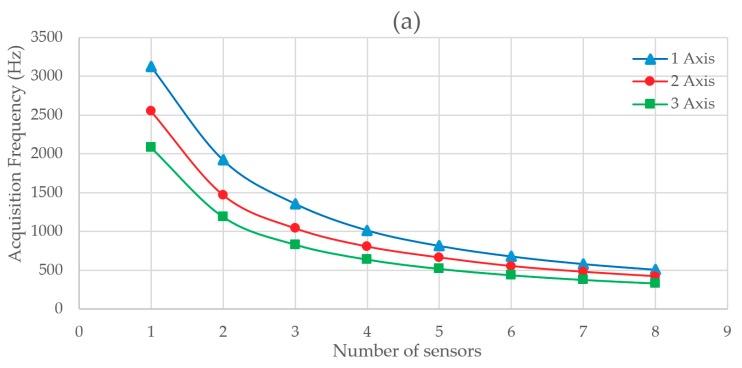

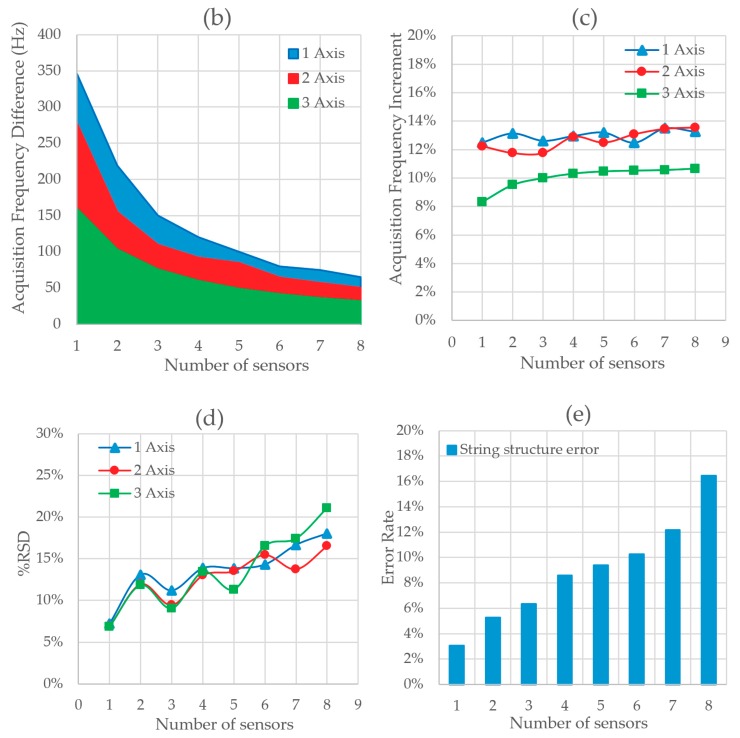
(**a**) Acquisition frequency depending on the number of sensors and axis read at the same time with Checksum method and 3 bytes of time, (**b**) total variation of the data acquisition frequency between Test 3 and Test 4, (**c**) percentage increase of the data acquisition frequency between Test 3 and Test 4, (**d**) acquisition frequency RSD depending on the number of sensors and axis read with 1 byte of time reduction, and (**e**) total error rate in Test 4.

Figure 10 shows the result of Test 4. Figure 10a shows the acquisition frequency for different numbers of sensors and axes at the same time. Figure 10b shows the absolute difference in the acquisition frequency between Tests 4 and 3, whereas Figure 10c shows the relative increase. There is an increase in the acquisition frequency thanks to the reduction of one byte of the time variable. In this case, the increment on the acquisition frequency achieved becomes linear with the number of sensors connected to the system. This new layer reduces one byte on the string for each sensor, and therefore the string is reduced in length proportionally to the number of sensors, in contrast with the previous layer, for which an extra checksum byte is included in the data string no matter the number of bytes that were being sent (number of sensors connected). Therefore, in that case, a logarithmic trend was found. The time needed to calculate the checksum is very small, and therefore its influence on the acquisition frequency is small. The acquisition frequency is mainly determined by the number of bytes sent. The standard deviation of the frequency is shown in Figure 10d where a variation between 7% and 20% is observed. The errors found are mostly short chain. These results are shown in Figure 10e.

**Figure 11:**
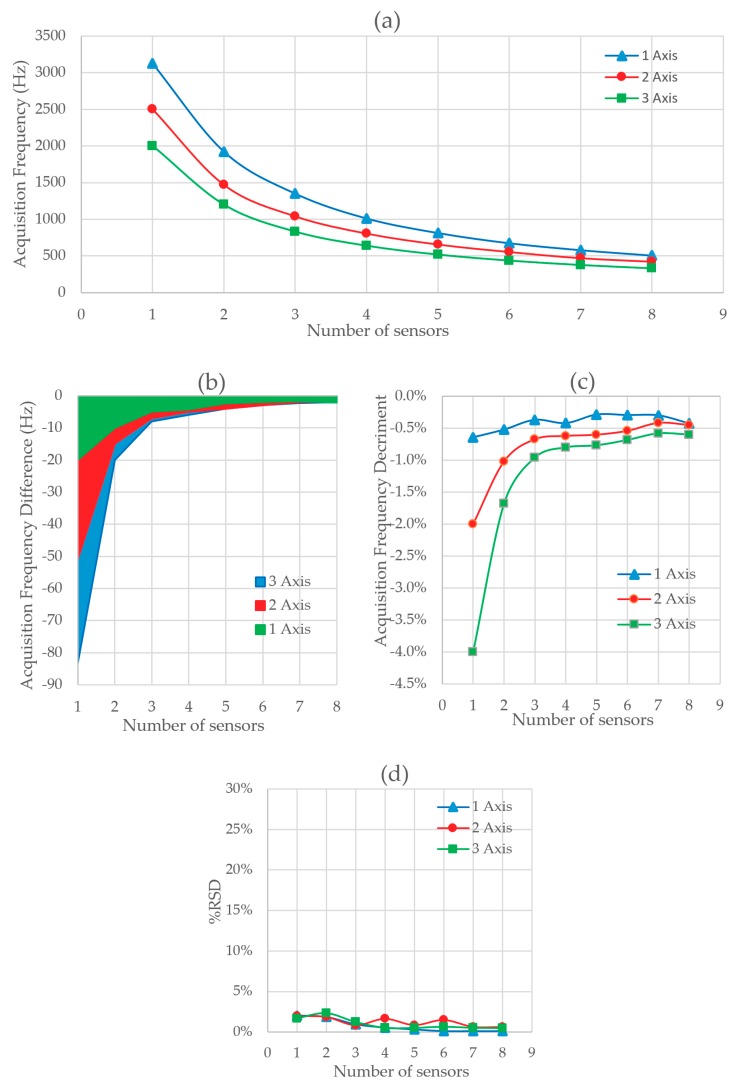
(**a**) Acquisition frequency depending on the number of sensors and axis read at the same time with stuffing method and 3 bytes of time, (**b**) total variation of the data acquisition frequency between Test 4 and Test 5, (**c**) percentage increase of the data acquisition frequency between Test 4 and Test 5, and (**d**) acquisition frequency RSD depending on the number of sensors and axis read with stuffing method and 1 byte of time reduction.

The results of Test 5 are shown in Figure 11. Figure 11a shows the absolute values of acquisition frequency versus the number of sensors and acquired axes. Figure 11b shows the absolute frequency difference between Test 5 and Test 4. It is observed that the frequency of acquisition is reduced moderately due to the stuffing calculation. As the number of bytes sent does not increase, there are no significant changes in the acquisition speed as compared with Test 4. Figure 11c shows the relative difference in the acquisition frequency between both tests. The relative deviation of the frequency is shown in Figure 11d. Deviation has been significantly reduced due to a decrease in errors. No errors were detected during the tests. We obtain a frequency of 3125 Hz for 1 MPU6050 sensor acquiring with an axis.

## 4. Discussion and Conclusions

**Figure 12:**
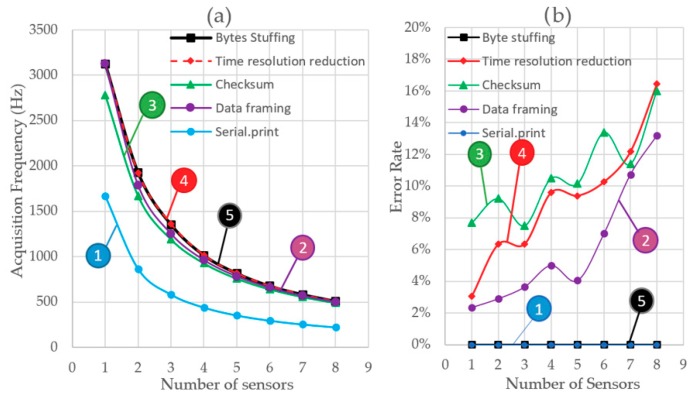

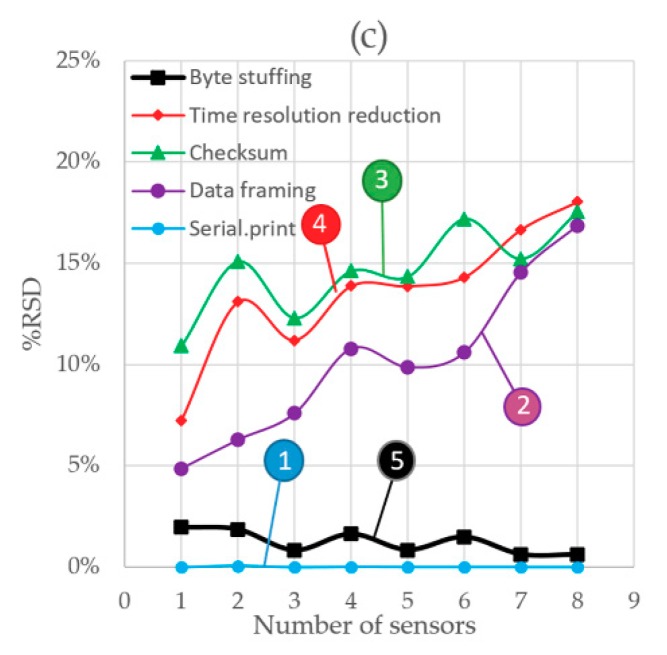
(**a**) Variation of acquisition frequency according to the method used, (**b**) variation of data transmission errors according to the method used, and (**c**) RSD of the acquisition frequency by method used.

Figure 12a shows the acquisition frequency variation for different number of sensors obtained when the different layers are included in the communication protocol. It can be observed that as soon as the Serial.print function (labeled as 1) is replaced by the data framing approach (labelled as 2) the sampling frequency is drastically increased. This is due, on one hand, to the fact that the number of bytes needed to transmit the information with the new approach is significantly reduced. On the other hand, the conversion of the actual value of a sensor‘s measure into the ASCII code transmitted by Serial.print is not needed anymore. Consequently, the time needed to send the information over the serial port is reduced.

When the second layer is introduced into the protocol, a checksum operation is performed in the side of the LC-DAQ and an extra byte is sent within the bytes’ string. The checksum operation increases the time that the microcontroller takes to perform a loop of its program and the extra byte increases the time needed to transmit the information. Both times impact negatively on the sampling frequency, as can be observed in the line labeled as 3. 

Up to this point, the resolution of the time variables was set as 32 bits (4 bytes). In order to improve the frequency, this resolution is reduced to 3 bytes. For each sensor measure, this implies that the number of bytes is reduced in one byte. Line number 4 shows how the sampling frequency is increased notably with this approach.

With the previous three layers, it can be observed that the number of errors produced on the serial communication increases with the number of sensors connected to the system (see Figure 12b). Similarly, as it is shown in Figure 12c, the variation in the frequency achieved tends to increase with the number of sensors. However, as soon as the stuffing layer is included in the protocol this trend disappears and the number of errors is reduced to practically zero. In the light of the results, it can be inferred that the vast majority of errors are due to the misinterpretation of one data byte as an end of transmission byte (ETX). Once the stuffing strategy is adopted the errors are fully avoided and the variation on the acquisition frequency is reduced, as shown in Figure 12c.

With the new communication protocol over the serial bus, including the four layers described in Section 2, higher acquisition frequencies can be obtained maintaining the robustness of the system. As a result of these improvements, a reliable low-cost data acquisition system that is capable of high acquisition frequencies is ready to be used in real-time vehicular dynamics. This system can be connected to a remote computer to transmit the data acquired in real time. The ready-to-use communication functions provided by a conventional low-cost platform such as Arduino present some drawbacks in terms of speed and error tracking in the communication process. The results presented in this paper depict a custom communication protocol that is able to increase the acquisition frequency by more than 200% as compared with that obtained by conventional protocols. It is based on serial bus and it has been built in different steeps. At each step a different layer is added and tested to observe the impact on the performance. Some layers aim to increase the communication speed, whereas other layers are implemented to guarantee the robustness and data consistency. Layers are added alternatively, increasing speed in one step and improving robustness in the next one. 

The standard deviation of the acquisition frequency is considered as a measure of the performance of the protocol in terms of robustness. Aleatory data losses imply larger variability on the time needed for a data string to be correctly delivered to the remote PC. Furthermore, the errors detected in the transmission are logged in order to identify which kinds of errors are more recurrent for each layer.

The new protocol is developed to send raw data bytes over the serial port in a framed structure which includes byte stuffing and 8 bits checksum. Furthermore, the resolution of time variables could be reduced to 24 bits by the inclusion of a time-correction routine that maintains the time coherence for virtual infinite measuring times. 

The systematic analysis of the performance of this new protocol shows that the amount of information sent over serial ports are the bottleneck on the acquisition process when low-cost platforms are used. Reducing the number of bytes on the data strings becomes crucial to increase the acquisition frequency. On the other hand, it was observed that the error with a higher recurrence received a byte string shorter than expected. It is produced when the stuffing layer was not implemented and a data byte with similar pattern of an ETX byte was mistaken by the serial reading program in the PC as the end of transmission sequence. However, once the stuffing layer was included, these errors were reduced to zero. 

Finally, an LC-DAQ acquisition system featuring this new protocol can double the acquisition frequency obtained with the baseline protocol. The number of errors in the transmission is reduced to practically zero and the variability on the communication times (acquisition frequency) takes very low values, similar to those obtained with the baseline protocol. Therefore, we conclude that this research supports the development of a powerful and suitable low-cost system for multisensor data acquisition in experiments where high frequency dynamics is involved.

The system, as it is currently conceived, needs to be connected to a computer for real-time acquisition applications. Further steps in the research are being taking in order to improve the system with a long-range wireless connection, and therefore a robust and high frequency capable telemetry system can be developed. For future research, we plan to analyze different technologies to replace the physical connection with the master computer with a wireless connection capable of operating over a long range (more than 500 m) with an acquisition frequency similar to that achieved in this investigation. These technologies could be based on 2.4 Ghz WiFi or LPWAN (low-power wide-area networks) more oriented to IoT [20], such as LoRa, NB-IoT, or Lte Cat-M1. 

## Figures and Tables

**Table 1:** Arduino Due characteristics.

Name	Arduino Due	Arduino Zero	Arduino Mega2560	Arduino Leonardo	Arduino Uno	Arduino Nano	Arduino Pro Mini
Format	Mega	Arduino	Mega	Arduino	Arduino	minimal	Mini
Processor	ATSAM3X8E (Cortex-M3)	ATSAMD 21G18A	Atmega 2560	Atmega 32U4	Atmega 328P	Atmega 328	Atmega 328P
Frequency	84 MHz	48 MHz	16 MHz	16 MHz	16 MHz	16 MHz	8 (3.3 V)
Digital I/O (pins)	54	14	54	20	14	14	14
Analog output pins	2	1	0	0	0	0	0
Analog input (pins)	12	6	16	12	6	8	6
Analog Signal Range	0–3.3 V	0–3.3 V	0–5 V	0–5 V	0–5 V	0–5 V	3.3 V
Analog signals resolution	12 bits	10 bits	10 bits	10 bits	10 bits	10 bits	10 bits
Analog signals resolution	0.81 mV	3.22 mV	4.88 mV	4.88 mV	4.88 mV	4.88 mV	3.22 mV

**Table 2:** LC-DAQ external connections.

Characteristic	Connection
Power	5 V, 3.3 V, 2Xgnd
Digital input/output	13
Analog inputs	12
Analog output	2
I2C	SDA 3.3 V, SCL 3.3 V, SDA 5 V, SCL 5 V
SPI	RESET, SCK, MISO, GND, MOSI, 5 V, 4 D I/O
LCD	5 V, GND, SDA 5 V, SCL 5 V
Others	IOREF, RESET

**Table 3:** Codification of special characters including their hexadecimal and binary values.

Byte	Hexadecimal	Binary
STX	0x02	0000 0010
ETX	0x03	0000 0011
ESC	0x06	0000 0110
